# Investigation of sumatriptan and ketorolac trometamol in the human experimental model of headache

**DOI:** 10.1186/s10194-020-01089-3

**Published:** 2020-02-24

**Authors:** Hashmat Ghanizada, Mohammad Al-Mahdi Al-Karagholi, Nanna Arngrim, Mette Mørch-Rasmussen, Matias Metcalf-Clausen, Henrik Bo Wiberg Larsson, Faisal Mohammad Amin, Messoud Ashina

**Affiliations:** 1 Danish Headache Center and Department of Neurology, Rigshospitalet Glostrup, Faculty of Health and Medical Sciences, University of Copenhagen, Valdemar Hansens Vej 5, DK-2600 Glostrup, Denmark; 2 Functional Imaging Unit, Department of Clinical Physiology, Nuclear Medicine and PET, Rigshospitalet, Faculty of Health and Medical Sciences, University of Copenhagen, Copenhagen, Denmark

**Keywords:** Headache, PACAP38, Mast cell degranulation, Plasma protein extravasation, Arterial dilation, Neuroinflammation, Pain, NSAIDs, MRA

## Abstract

**Background:**

Pituitary adenylate cyclase-activating polypeptide-38 (PACAP38) induces headache in healthy volunteers but the precise mechanisms by which PACAP38 leads to headache are unclear. We investigated the headache preventive effect of sumatriptan and ketorolac on PACAP38-induced headache in healthy volunteers. In addition, we explored contribution of vascular mechanisms to PACAP38-induced headache using high resolution magnetic resonance angiography.

**Methods:**

Thirty-four healthy volunteers were divided in two groups (A and B) and received infusion of PACAP38 (10 picomol/kg/min) over 20 min. Group A was pretreated with intravenous sumatriptan (4 mg) or ketorolac (30 mg) 20 min before infusion of PACAP38. Group B received infusion of sumatriptan or ketorolac as post-treatment 90 min after infusion of PACAP38. In both experiments, we used a randomized, double-blind, cross-over design. We recorded headache characteristics and circumference of extra-intracerebral arteries.

**Results:**

We found no difference in AUC _(0–6 h)_ of PACAP38-induced headache in group A, pretreated with sumatriptan or ketorolac (*p* = 0.297). There was no difference between sumatriptan and ketorolac in PACAP38-induced circumference change (AUC_Baseline-110 min_) of MMA (*p* = 0.227), STA (*p* = 0.795) and MCA (*p* = 0.356). In group B, post-treatment with ketorolac reduced PACAP38-headache compared to sumatriptan (*p* < 0.001). Post-treatment with sumatriptan significantly reduced the circumference of STA (*p* = 0.039) and MMA (*p* = 0.015) but not of MCA (*p* = 0.981) compared to ketorolac. In an explorative analysis, we found that pre-treatment with sumatriptan reduced PACAP38-induced headache compared to no treatment (AUC_0-90min_).

**Conclusions:**

Post-treatment with ketorolac was more effective in attenuating PACAP38-induced headache compared to sumatriptan. Ketorolac exerted its effect without affecting PACAP38-induced arterial dilation, whereas sumatriptan post-treatment attenuated PACAP38-induced dilation of MMA and STA. Pre-treatment with sumatriptan attenuated PACAP38-induced headache without affecting PACAP38-induced arterial dilation. Our findings suggest that ketorolac and sumatriptan attenuated PACAP38-induced headache in healthy volunteers without vascular effects.

**Trial registration:**

Clinicaltrials.gov (NCT03585894). Registered 13 July 2018,

## Background

Pituitary adenylate cyclase-activating polypeptide 38 (PACAP38) is a pleiotropic signaling neuropeptide [33, 34] that induces headache in healthy volunteers and migraine attacks in migraine patients [4, 42]. PACAP38 is located in both sensory and parasympathetic perivascular nerve fibers [35, 52] and its infusion causes prolonged extracerebral dilation [5, 7, 13, 20, 49] and dural mast cell degranulation [12]. Recent data showed that PACAP38 activated mast cell specific receptor Mas-related G-protein-coupled receptors-b2 (Mrgprb2) [22, 38] which mediates neurogenic inflammation and pain [22]. Activation of mast cells leads to recruitment of dural immune cells involving neutrophils, monocytes and macrophages [25, 40, 47]. Dural neurogenic inflammation and mast cell mediated activation of the trigeminal pain pathway have been suggested to play a key role in migraine pathogenesis [31, 36]. The precise mechanisms by which PACAP38 leads to headache and migraine are unclear.

The anti-migraine-specific drug sumatriptan, a 5-HT_1B /1D_ agonist [18], is a vasoconstrictor [6, 27] with anti-inflammatory properties [16] that potently blocks neurogenic plasma extravasation from dural blood vessels [17]. Ketorolac is cyclooxygenase (COX-1 and COX-2) inhibitor non-steroidal anti-inflammatory drug [41, 44, 48] that reduces mast cell degranulation [50] and blocks dural macrophage activation [37]. The neurovascular effects of ketorolac have not been studied in humans. Sumatriptan and ketorolac are used as abortive medication for migraine treatment but the site and mode of action of these drugs are not fully clarified.

In the present study, we used PACAP38 as a biomarker of headache with inflammatory and vascular components. To further elucidate the mechanisms underlying the action of sumatriptan and ketorolac, we investigated the effect of both drugs on PACAP38-induced headache in healthy volunteers. We hypothesized that both sumatriptan and ketorolac would attenuate PACAP38-induced headache but only sumatriptan infusion would abolish PACAP38-induced arterial dilation. To test this hypothesis we conducted a randomized, double-blind, crossover study and used magnetic resonance angiography (MRA) to record vascular responses.

## Methods

### Participants

We recruited thirty-four healthy volunteers. All participants were pre-screened over telephone and all potential study candidates were invited to the hospital for thorough screening. The eligibility criteria for inclusion in the study were as follows; adults ≥18 to ≤50 years of age of both sexes with body weight of 50 to 100 kg. Exclusion criteria included: daily intake of any medication except contraceptives, magnetic resonance imaging contraindications, serious somatic disease (including any pain condition), history of migraine or any other type of headache expect episodic tension-type headache less than once a month. All participants provided detailed oral and written information about the study and written informed consent was obtained in accordance with the Helsinki declarations. The study was approved by the Ethics Committee of the Capital Region of Denmark (H-18008313) and registered at Clinicaltrials.gov (ID: NCT03585894).

### Experimental design

**Fig. 1:**
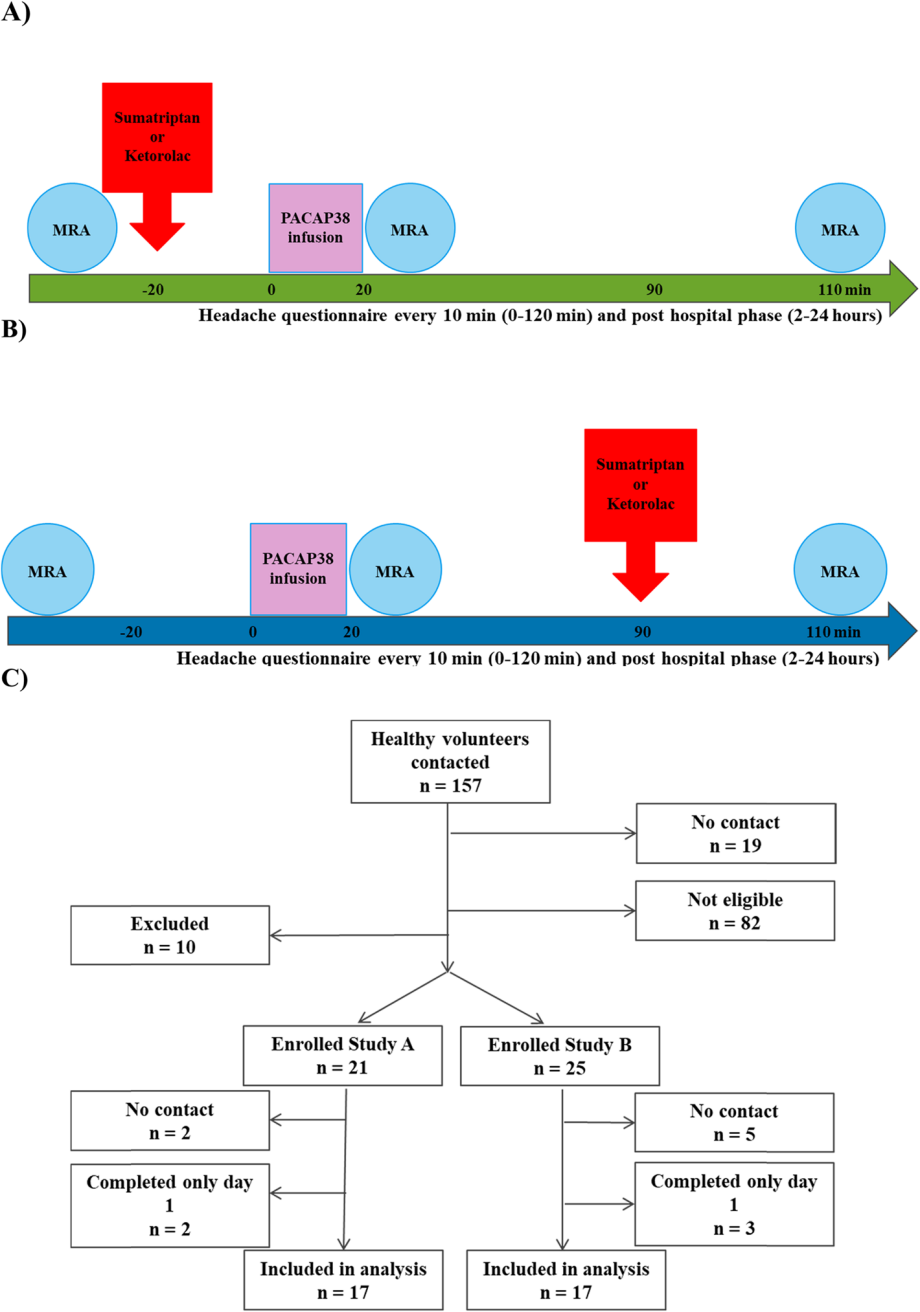
**a** Pre-treatment study design. **b** Post-treatment study design. **c** Recruitment flow chart. All participants took part in each group in two experimental days separated by one week

We divided participants into two groups: group A and group B. In group A, participants were randomly assigned to intravenous infusion of sumatriptan 4 mg (GlaxoSmithKline Pharma A/S, Denmark) or ketorolac trometamol 30 mg (Atnahs Pharma, UK Limited) over 10 min. At 20 min after start of infusion of sumatriptan and ketorolac participants received infusion of PACAP38 (10 picomole/kg/min) [42] over 20 min (Fig. 1 a). In group B, participants first received PACAP38 infusion over 20 min and at 90 min after start of infusion randomly assigned to receive infusion of sumatriptan or ketorolac (Fig. 1 b). In each group, experiments were conducted on two separate days with a washout period of one week. The PACAP38 (Bachem) solution was prepared for the study by the Capital Region Hospital Pharmacy.

All participants were non-fasting and arrived at the hospital 24 h headache free and were instructed not to consume coffee, tea, cola, caffeine, alcohol, smoking and avoid exhausting physical activities for 12 h prior to the study day. On arrival, female participants were tested for pregnancy on both days and a venous catheter (Venflon, Becton Dickinson Infusion Therapy AB, Sweden) was inserted into the antecubital vein for infusion. After 15 min of rest in the supine position, headache intensity and characteristics, vital signs, mean arterial blood pressure (MAP), and heart rate (HR) were recorded at baseline and at fixed intervals every 10 min (0–120 min). All participants underwent three MRI sessions: baseline, 20 min and 110 min (Fig. 1). All experiments were conducted in the afternoon due to limited availability of the MRI scanner. We were therefore only able to record headache data from all participants from 0 to 6 h since most participants went to sleep thereafter.

### Headache

We used a validated headache questionnaire to record headache intensity using numerical rating scale from 0 to 10 (0: no headache; 1: a very mild headache (including a feeling of pressing or throbbing pre-pain); 10: worst imaginable headache) and associated symptoms. Headache characteristics (localization, quality, aggravation by physical activity, associated symptoms (nausea, photo- and phonophobia)) and prodromes (unusual fatigue, yawning, thirst, craving, mood swings, flushing and difficulty concentrating) were also recorded.

### Data acquisition and image analysis

All scans were performed on a Philips 3 T Achieva MRI scanner (Philips Medical Systems, Best, The Netherlands) using a 32-channel phase array head coil. A 3D time-of-flight MRA of extra-intracerebral arteries was acquired as described in previous studies [5, 6]. All acquired MRA data were saved in DICOM format and transferred to a separate workstation and analyzed by LAVA-MRA vessel wall analysis software program. LAVA-MRA was previously used in several studies [4, 8] and the method demonstrated a low < 5% inter- and intra-observer variation [3]. The software automatically detects the vessel contours and calculate the circumference every 0.2 mm perpendicular to the center line. For each vessel an average of 26 slides (5 mm) was obtained and the measurement was repeated for all participant at the same vessel segment.

Two bilateral branches of superficial temporal artery (STA), middle meningeal artery (MMA) and middle cerebral artery (MCA) were analyzed by an investigator who was blinded to the experimental day and scan session.

### Statistical analysis

All absolute values are presented as mean with 95% confidence interval (CI). We calculated the study sample size based on headache and vascular responses to PACAP38 taking the previous findings into consideration [5]. The risk of type 1 error at 5% and a defined power at 80% and type 2 error was fixed at 20%. We calculated that thirty-four participants would be adequate for a crossover study design.

The primary endpoints of the study were a difference in the area under the curve (AUC) for headache (0–6 h) and arterial circumference change of STA, MMA and MCA from baseline to 110 min between treatment with sumatriptan and ketorolac. The secondary endpoints were difference in AUC for HR (0–120 min), MAP (0–120 min) and associate symptoms (0–24 h). Baseline values were subtracted before calculating AUC to reduce within-participant variation between sessions. The non-parametric Wilcoxon signed rank test was used to calculate the side to side difference in arterial circumference. We used Mann-Whitney test to compare the differences between the groups.

All statistical analyses were performed with SPSS version 23.0 (Chicago, IL, USA). We did not adjust for multiple comparisons, as our primary endpoints, hypotheses and statistical tests were all predefined and clearly stated in the study protocol. Five percent (*p* < 0.05) was accepted as the level of significance.

## Results

Thirty-four healthy volunteers (18 females and 16 males, mean age 25 years, (range 19–39 years) and mean weight of 70 kg, (range 54–88 kg)) completed the study. PACAP38 infusion induced headache in sixteen out of seventeen (94%) subjects pre-treated (10 females and 7 males) with ketorolac and thirteen out of seventeen (77%) pre-treated with sumatriptan. In post-treatment group (8 females and 9 males), PACAP38 infusion induced headache in sixteen out of seventeen (94%) treated with ketorolac and seventeen out of seventeen (100%) treated with sumatriptan. (Table 1).

**Table 1:** Headache incidence and characteristics after PACAP38 infusion and treatment with ketorolac and sumatriptan from 0 to 2 and 2–6 h. Headache inductions rate: number of participants who developed headache; Median peak headache: peak headache intensity recorded on a numerical rating scale from 0 to 10

PACAP38 infusion/treatment	0–2 h Headache induction rate	2–6 h Headache induction rate	0–2 h Median Peak headache (range)	2–6 h Median Peak headache (range)	0–2 h Median Duration of headache (hours)	2–6 h Median Duration of headache (hours)
Pre-treatment Ketorolac	**16 of 17**	**13 of 17**	**1 (0–5)**	**0 (0–3)**	**1**	**2**
Pre-treatment Sumatriptan	**13 of 17**	**8 of 17**	**1 (0–4)**	**0 (0–5)**	**0.17**	**1**
Post-treatment Ketorolac	**16 of 17**	**12 of 17**	**1 (0–5)**	**1 (0–2)**	**1.17**	**4**
Post-treatment Sumatriptan	**17 of 17**	**12 of 17**	**2 (0–7)**	**1 (0–4)**	**1.67**	**2**

We found no difference in arterial circumferences and vital variables at baseline between sumatriptan and ketorolac day in both study groups (A and B). There was no carry-over or period effect for baseline values between the study days. We found no difference between the right and left sided arteries (*p* > 0.05) and therefore, an average of both arteries was used.

### Effect of pre-treatment of sumatriptan or ketorolac: group A

**Fig. 2:**
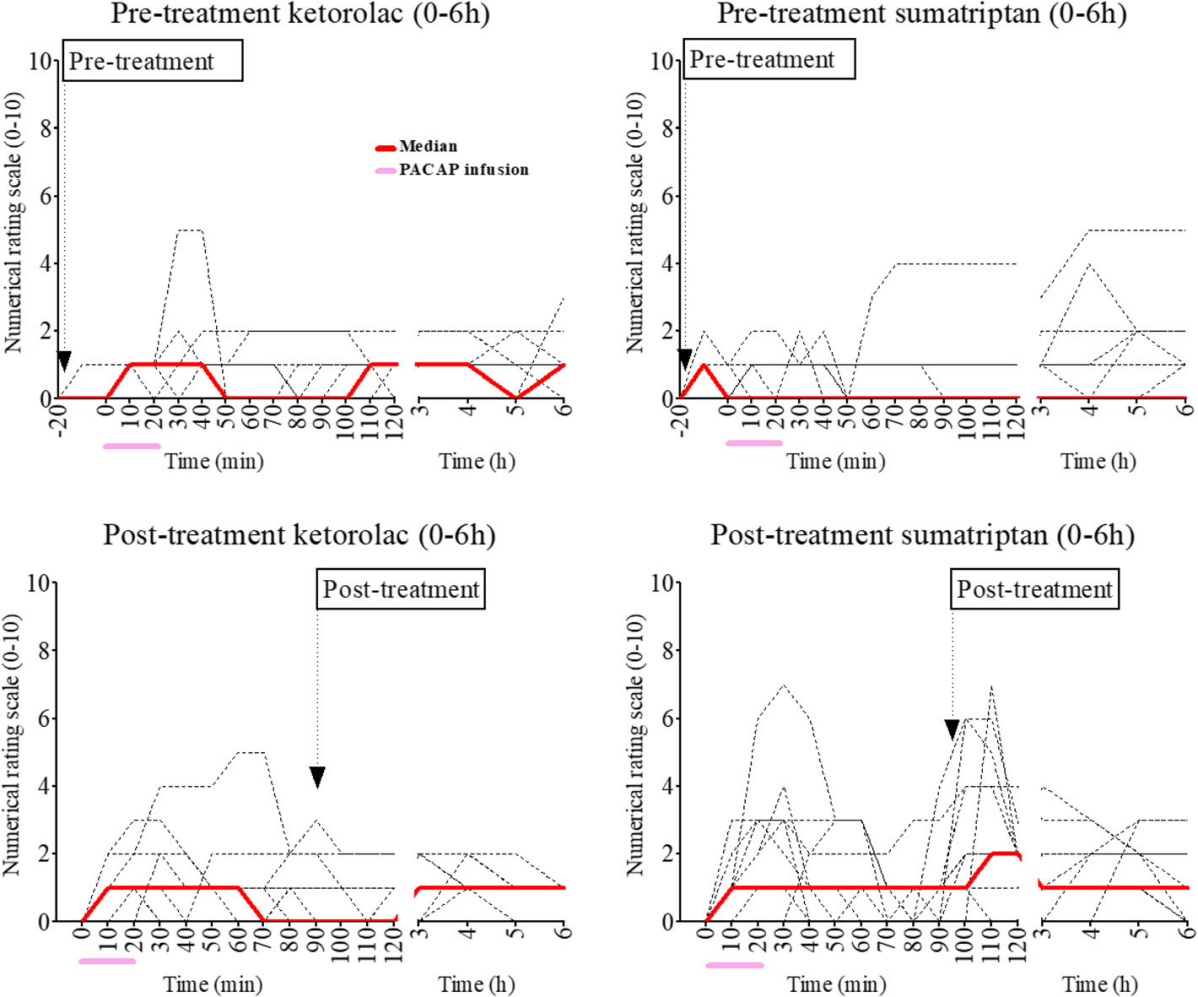
Headache intensity of individual volunteers (black lines) and the median headache intensity (red line) after PACAP38 and pre-treatment with ketorolac /sumatriptan (*n* = 17) and post-treatment with ketorolac /sumatriptan (n = 17)

**Fig. 3:**
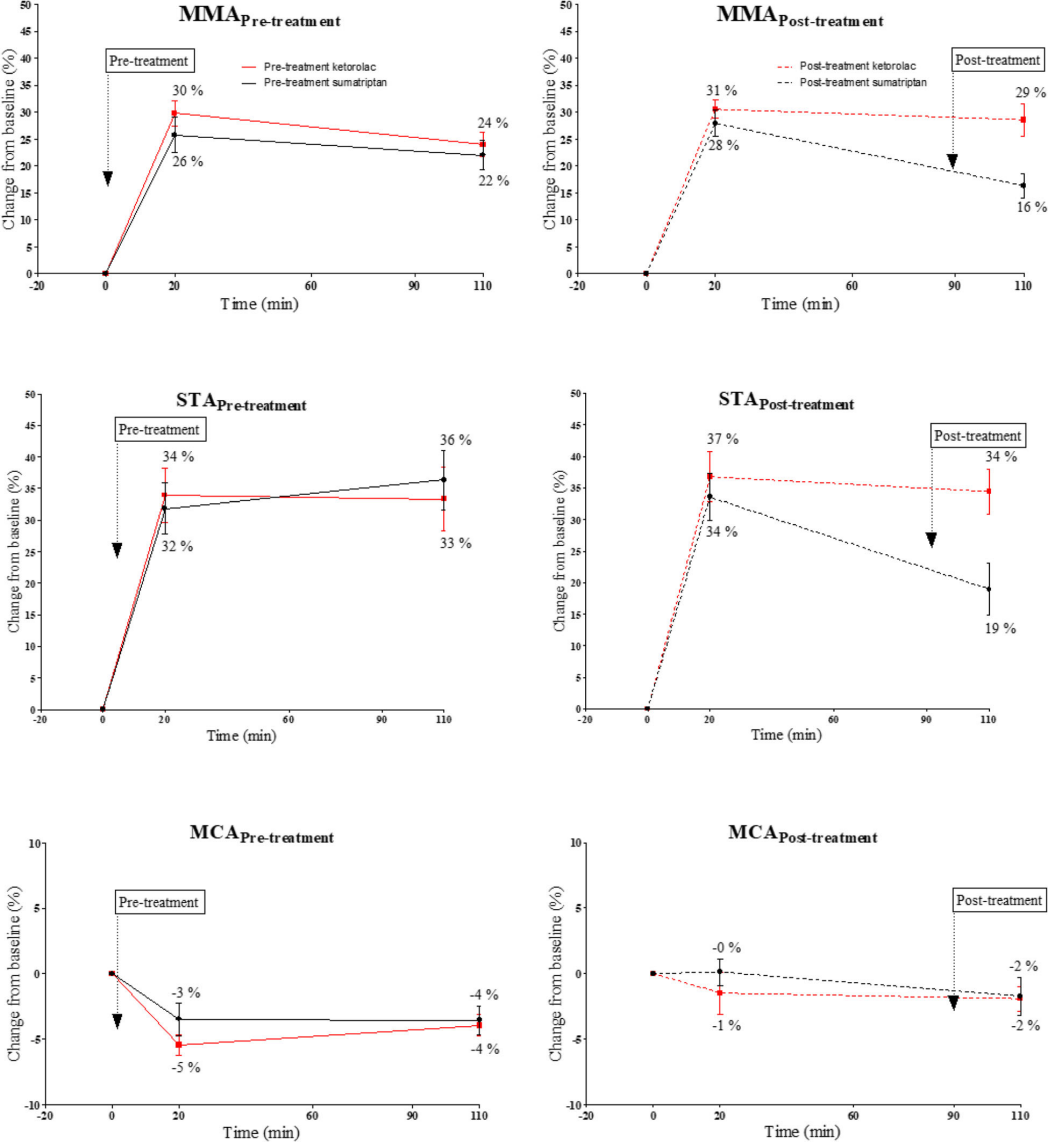
Effect of pre-treatment (solid line) and post-treatment (dotted line) on extra-intracerebral arteries dilated with PACAP38. Middle meningeal artery (MMA), superficial temporal artery (STA) and middle cerebral artery (MCA)

We found no difference in AUC _(0–6 h)_ for PACAP38-induced headache between sumatriptan and ketorolac (*p* = 0.297) (Fig. 2, Table 1). There was no difference in PACAP38-induced circumference change (AUC_Baseline-110 min_) of MMA (*p* = 0.227), STA (*p* = 0.795) and MCA (*p* = 0.356) after sumatriptan compared to ketorolac (Fig. 3).

### Effect of post-treatment of sumatriptan or ketorolac: group B

We found that ketorolac reduced the AUC _(1.5–6 h)_ for PACAP38-induced headache compared to sumatriptan (*p* < 0.001) (Fig. 2, Table 1). Sumatriptan significantly reduced the circumference of STA (*p* = 0.039) and MMA (*p* = 0.015) compared to ketorolac. No difference was found in circumference change of MCA (*p* = 0.981) (Fig. 3).

### Explorative analyses on headache data

To explore whether both drugs prevent PACAP38-induced headache we conducted an explorative analysis on headache data between group A and B from 0 to 90 min: pretreatment with sumatriptan or ketorolac followed by PACAP38 infusion compared to group B where participants received only PACAP38 infusion up to 90 mins. The AUC _(0–90 min)_ for headache score was significantly larger after PACAP38 infusion compared to PACAP38-induced headache pre-treated with sumatriptan (*p* = 0.005). A trend of attenuation of PACAP38-induced headache was recorded in those who were pre-treated with ketorolac compared to only PACAP38 infusion (*p* = 0.076) (Fig. 2).

### Vital variables and adverse events

**Fig. 4:**
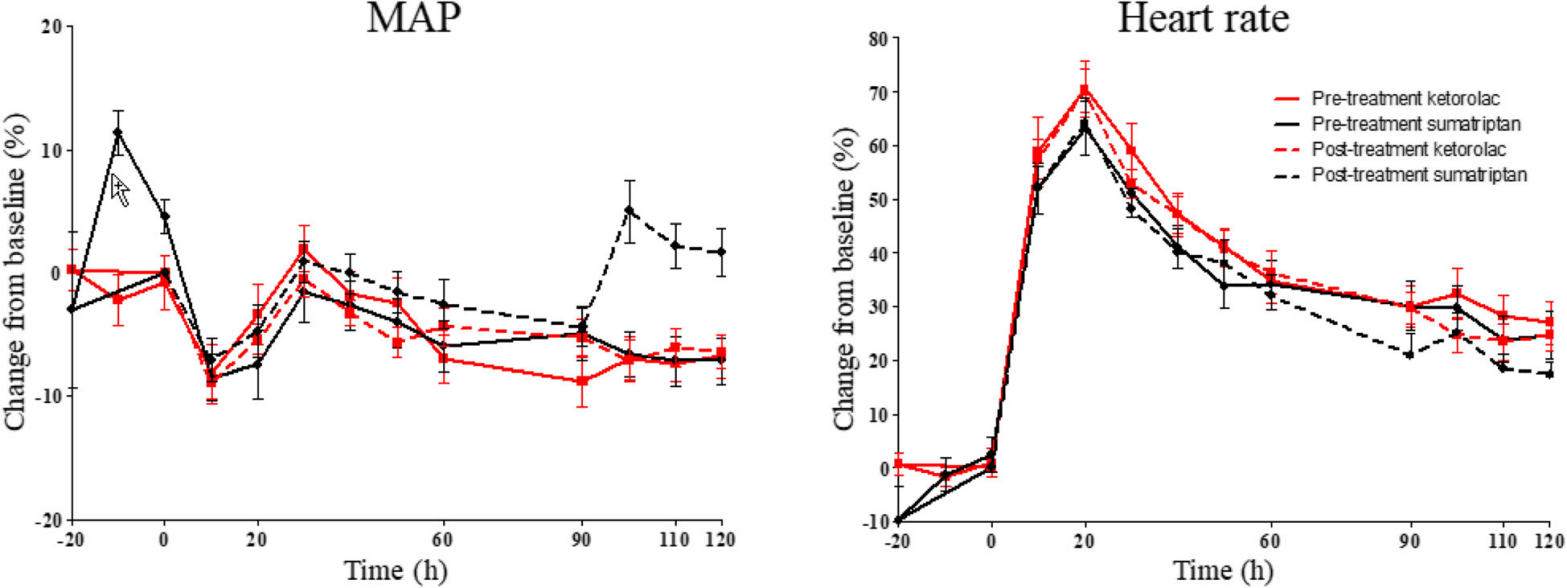
Percentage change from baseline for mean arterial pressure (MAP) and heart rate (HR) after PACAP38 and pre-treatment and post-treatment with ketorolac and sumatriptan

In pre-treatment group A, we found no difference in AUC _(Baseline-120 min)_ for MAP changes between ketorolac and sumatriptan (*p* = 0.523). In post-treatment group B, the AUC _(Baseline-120 min)_ for MAP was significantly larger after sumatriptan compared to ketorolac (*p* = 0.028). We found no change in AUC _(Baseline-120 min)_ for heart rate in pre-treatment group between sumatriptan and ketorolac (*p* = 0.492) and post-treatment group between sumatriptan and ketorolac (*p* = 0.356) (Fig. 4).

There was no difference in incidence of nausea, difficulty concentrating, neck stiffness, fatigue, thirst and facial puffiness (Table 2).

**Table 2:** Adverse events after PACAP38 infusion and treatment with ketorolac and sumatriptan

Variables	Pre-treatment (Ketorolac)	Pre-treatment (Sumatriptan)	Post-treatment (Ketorolac)	Post-treatment (Sumatriptan)
Palpitation	100%	94.1%	100%	100%
Flushing	100%	100%	100%	100%
Heat sensation	100%	100%	100%	100%
Nausea	0%	0%	0%	0%
Neck stiffness	11.8%	11.7%	17.7%	17.7%
Difficulty concentrating	5.9%	5.9%	23.5%	17.8%
Fatigue	23.5%	17.7%	23.5%	23.5%
Thirst	17.7%	11.8%	11.8%	23.5%
Facial puffiness	41.2%	35.3%	41.2%	17.7%

## Discussion

The main findings of the present study were: 1) Participants who were pre-treated with sumatriptan or ketorolac reported no difference in PACAP38-induced headache; 2) Post-treatment with ketorolac was more effective in attenuating PACAP38-induced headache compared to sumatriptan. Interestingly, the ketorolac exerted its effect without affecting PACAP38-induced arterial dilation, whereas sumatriptan post-treatment attenuated PACAP38-induced dilation of MMA and STA. Explorative analysis showed that pre-treatment with sumatriptan attenuated PACAP38-induced headache without affecting PACAP38-induced arterial dilation. In the current study both drugs given as pre- or post-treatment had no effect on PACAP38-induced autonomic symptoms such as palpitation, facial flushing and heat sensation. Most participants reported facial flushing and puffiness persisting over 24 h post PACAP38 infusion. PACAP is found in human skin [53] and PACAP infusion causes intense facial flushing [21].

### Experimentally induced headache and anti-migraine medication

Similar to previous studies [5, 42], we found that PACAP38 infusion caused prolonged mild to moderate headache in 90% of healthy volunteers. The headache preventive effect of sumatriptan when given before PACAP38 infusion is consistent with previous studies demonstrating that pre-treatment with sumatriptan reduced glycerol trinitrate (GTN) [28] and cilostazol [19] induced headache in healthy volunteers. GTN is a pro-drug for nitric oxide (NO) and cilostazol is a phosphodiesterase 3 inhibitor which works downstream in the cascade of events associated to headache [19, 28]. It has been suggested that sumatriptan prevents headache in these models by inhibiting accumulation of cyclic guanosine monophosphate (cGMP) and cyclic adenosine monophosphate (cAMP). Interestingly, post-treatment with triptans failed to antagonize GTN and cilostazol induced headache in healthy volunteers [23, 46]. In line with this, we also show that post-treatment with intravenous administration of sumatriptan failed to prevent PACAP38-induced headache in healthy volunteers. The failure of sumatriptan to abort PACAP38-induced headache might be due to establishment of central sensitization and disruption of presynaptic 5-HT_1B /1D_ receptors in the dorsal horn [32].

The lower AUC for headache score (2–6 h) after ketorolac compared to sumatriptan post-treatment suggests that ketorolac was more effective than sumatriptan when administered as post-treatment during the established PACAP38-induced headache phase in healthy volunteers. Our findings are consistent with previous studies demonstrating that ketorolac infusion is able to terminate established peripheral and central sensitization [29]. However, our results should be interpreted with caution because sumatriptan infusion caused immediate but short exacerbation of PACAP38-induced headache. The sumatriptan-induced headache was previously observed after infusion of calcitonin gene-related peptide (CGRP), levcromakalim, isosorbide-5-mononitrate (NO donor) and cilostazol in healthy volunteers [2, 9, 23, 24]. It is also a well-known side effect of sumatriptan treatment in migraine patients [15, 39]. This may explain the difference between the two drugs administered as post-treatment. However, the similar incidence of headache (Table 1) and unchanged median headache score (Fig. 2) for both drugs in post-hospital phase indicates that both drugs are not effective in aborting PACAP38-induced headache during this phase.

### Cranial artery dilation and anti-migraine medication

Similar to previous studies [4, 5], we found that PACAP38 infusion caused sustained dilation of extracerebral arteries but not intracerebral arteries. Though earlier studies have shown that sumatriptan constricts extracerebral arteries [5, 6], sumatriptan pre-treatment was unable to counteract PACAP38-induced dilation of MMA and STA. This result shows that PACAP38 bypass the vasoconstrictive effect of sumatriptan in these arteries in healthy volunteers. The headache preventive effect of sumatriptan given before PACAP38 infusion without affecting the vasodilatory response to PACAP38 suggest that the headache inducing effect of PACAP38 is independent of its vasoactive property in healthy volunteers.

In the present study, we found that post-treatment with sumatriptan but not ketorolac reduced MMA and STA circumference, but the dilation of MMA and STA did not return to baseline during the observation period. In humans, sumatriptan constricts normal and pre-dilated extracerebral arteries [5, 6, 10]. In healthy volunteers, subcutaneous injection of sumatriptan totally abolished a modest CGRP induced dilation of MMA [11]. Taken together, it seems that PACAP38-induced vasodilation mechanistically differs from CGRP and its prolonged dilation of extracerebral arteries might be caused via activation of dural mast cells [12]. Identification of a putative new PACAP-receptor on mast cells provides important insight on PACAP38-induced prolonged dilation of extracerebral arteries [38]. Dural mast cells are found in close proximity to meningeal nociceptors, whereupon activation releases vasoactive neuropeptides which mediates activation of pain pathways [22].

To the best of our knowledge, no study investigated possible vasoactive properties of ketorolac using advanced MRA method. Our data demonstrated that ketorolac had no vascular effect on extra-intracerebral arteries and neither pre nor post-treatment alters the vascular effects of PACAP38. It is possible that pro-inflammatory prostanoids are released by PACAP38 induced mast cell degranulation [12, 30, 45] and the headache attenuating effect of ketorolac treatment might be via inhibition of pro-inflammatory prostanoids activated by PACAP38 infusion.

### Mechanisms behind the anti-nociceptive effect of sumatriptan and ketorolac

It has been reported that PACAP38 induces sensitization of trigeminal neurons via activation of neuronal PAC_1_ receptor [1]. In animals, pre-treatment with sumatriptan effectively blocked the development of all aspects of central sensitization by blocking the peripheral signal transmission from the meningeal nociceptors [14]. Moreover, it has been shown that sumatriptan can inhibit trigeminal activation without its vasoconstrictive effects [26]. It has been postulated that sumatriptan exerts its antinociceptive effect by disrupting communication between peripheral and central trigeminovascular neurons [14]. In the current study, we showed that pre-treatment with sumatriptan was more effective in attenuating PACAP38-induced headache. Ketorolac may exert its effect by suppression of central sensitization by directly silencing the peripheral and central trigeminovascular neurons [29]. Ketorolac treatment was more effective in terminating headache and allodynia in migraine patients who had established central sensitization [29]. Interestingly, we found that pre-treatment with ketorolac failed to prevent PACAP38-induced headache, though as mentioned a trend was observed. We suggest that the antinociceptive effect of ketorolac may partly depend on a prior activation of pro-inflammatory prostanoids.

PACAP38 degranulates dural mast cells and this mechanism may mediate PACAP38-induced prolonged arterial dilation [12]. Recent studies reported that PACAP38 caused degranulation of mast cells and histamine release via a specific receptor Mrgprb2 [22] which leads to prolonged activation of the trigeminal pain pathway [31]. In animals, sumatriptan inhibits mast cell degranulation [31], potently blocks neurogenic plasma protein extravasation from dural blood vessels [17] and prevents release of neuropeptides from perivascular neurons [16, 17]. As a COX inhibitor ketorolac exerts its analgesic and anti-inflammatory effects via depression of prostanoid biosynthesis [43]. Prostaglandins and their receptors are widely distributed in the extra-intracerebral arteries, trigeminal ganglion and trigeminal nucleus caudalis [43]. In addition to histamine dural mast cells can release various inflammatory mediators including prostaglandin I_2_ that can activate and sensitize meningeal sensory afferents [51].

Taken together, the current study revealed that sumatriptan pre-treatment was more effective in preventing PACAP38-induced headache. This suggests that sumatriptan may have time dependent preventative properties which require further investigation.

## Conclusion

The major finding of the present study was that no difference reported in PACAP38-induced headache after pre-treatment with sumatriptan or ketorolac. We found that post-treatment with ketorolac was more effective in attenuating PACAP38-induced headache compared to sumatriptan. Ketorolac exerted its effect without affecting PACAP38-induced arterial dilation, whereas sumatriptan post-treatment attenuated PACAP38-induced dilation of MMA and STA. Explorative analysis showed that pre-treatment with sumatriptan attenuated PACAP38-induced headache without affecting PACAP38-induced arterial dilation.

## Data Availability

Anonymized data can be shared, until one year after publication, upon request to the corresponding author from qualified investigators for purposes of replicating procedures and results.

## Abbreviations

AUC: Under the curve
cAMP: Cyclic adenosine monophosphate
cGMP: Cyclic guanosine monophosphate:
CGRP: Calcitonin gene-related peptide
CI: Confidence interval
COX: Cyclooxygenase
GTN: Glycerol trinitrate
HR: Heart rate
MAP: Mean arterial blood pressure
MCA: Middle cerebral artery
MMA: Middle meningeal artery
MRA: Magnetic resonance angiography
Mrgprb2: Mas-related G-protein-coupled receptors-b2
NO: Nitric oxide
PACAP38: Pituitary adenylate cyclase-activating polypeptide-38
STA: Superficial temporal artery
